# Environmental factors affecting akinete germination and resting cell awakening of two cyanobacteria

**DOI:** 10.1186/s42649-023-00085-6

**Published:** 2023-01-17

**Authors:** Daeryul Kwon, Keonhee Kim, Hyunjin Jo, Sang Deuk Lee, Suk Min Yun, Chaehong Park

**Affiliations:** 1 Protist Research Team, Microbial Research Department, Nakdonggang National Institute of Biological Resources (NNIBR), 137, Donam 2-Gil, Sangju-Si, 37182 Korea; 2 ZION E&S CO., Ltd., Pentaplex, 66, Daehwa-Ro 106Beon-Gil, Daedeok-Gu, 1133 Daejeon, Republic of Korea

**Keywords:** Akinete, Resting cell, *Dolichospermum*, *Microcystis*

## Abstract

Globally, cyanobacteria frequently cause blooms that outcompete other species in the waterbody, affecting the diversity, decreasing water exchange rates, and promoting eutrophication that leads to excessive algal growth. Here, *Dolichospermum circinale* (akinetes) and *Microcystic aeruginosa* (resting cells), were isolated from the sediment in the Uiam Dam in the North Han River and near Ugok Bridge in the Nakdong River, respectively. The morphology, germination process and rates, and growth was evaluated in different environmental conditions. *D. cercinalis* germination began on day two of culturing, with maximum cell growth observed on day ten. In contrast, *M. aeruginosa* exhibited daily increase in cell density and colony size, with notable density increase on day six. Next, different environmental conditions were assessed. Akinetes exhibited high germination rates at low light intensity (5—30 µmol/m^2^/s), whereas resting cells exhibited high growth rates at high light intensity (50—100 µmol/m^2^/s). Furthermore, both cell types exhibited optimum germination and growth in media containing N and P at 20—30° at a pH of 7—9. Our study reveals the optimum conditions for the germination and growth of cyanobacterial akinetes and resting cells isolated from river sediment, respectively, and will assist in predicting cyanobacterial blooms for appropriate management.

## Introduction

Cyanobacteria are known to frequently cause blooms in eutrophic lakes and rivers worldwide (Dokulil and Teubner 2000; Paerl and Otten 2013). The phytoplankton density increases as the water temperature, light intensity, and nutrient salts increase in the eutrophic lakes and rivers, and cyanobacteria (among phytoplankton) establish dominance that lead to a higher probability of blooms (Sommer 1985; Tryfon and Moustaka-Gouni 1997). To adapt in the waterbody, cyanobacteria outcompete other species through seasonal lifecycle patterns (Visser et al. 1997). Owing to their characteristic floating capability, the environmental conditions are favorable to cyanobacteria over other algal species in summer, and they obtain a competitive edge regarding temperature, light, and nutrient consumption (Walsby 1994; Paerl and Fulton 2006). However, certain cyanobacteria species produce toxic substances or harmful taste and odor compounds to cause problems related to the ecosystem health and public health (Wert et al. 2014; Harris and Graham 2017). The blooms caused by cyanobacteria are attributed to factors that increase the water temperature and daylight, decrease the water exchange rate, and promote eutrophication that fundamentally induces algal growth, as well as algal cell accumulation and variation in and out of the waterbody due to hydraulic and hydrologic factors such as draught and increased retention time (Liu et al. 2007; Pick 2016). The species-specific life history within the ecosystem presents a basic premise that allows a deeper understanding regarding cyanobacteria (Park et al. 2018). Cyanobacteria are detected worldwide, especially in summer with increased water temperatures (Sigee et al. 2007; Havens 2008). However, unprecedented cyanobacteria blooms have occurred in recent winters (Üveges et al. 2012; Wejnerowski et al. 2018). Recent studies reported that the dominant species of cyanobacteria varies according to the water system or region, while the known harmful species of the *Dolichospermum* and *Microcystis* genera cause blooms in summer, and the species of the *Aphanizomenon* genus cause blooms mainly in autumn (Yamamoto and Nakahara 2005; Wu and Kong 2009; Li et al. 2016). These blooms are caused by increased water temperature, sunlight, draught, and eutrophication (Paul 2008; Carey et al. 2012). The Han River water system, in particular, shows the periodic dominance by the *Dolichospermum* genus of the order Nostocales (Byun et al. 2015; Choi et al. 2018; Kim et al. 2020). In contrast, the Nakdong River water system is mainly dominated by the *Microcystis* genus (Jeong et al. 2003; Ha et al. 1999). The survival strategy of cyanobacteria consists of the formation of special akinetes (Nostocales and Stigonematales) and resting cells (Chroococcales) (Hirose et al. 1977; Hindák 2000; Cirés et al. 2013). The main environmental factors that regulate the germination process of akinetes and resting cells are water temperature, light status, pH, and nutrients, and these optimal conditions may vary across species (Huber 1985; Kremp and Anderson 2000; Kim et al. 2005). The survival strategy of cyanobacteria based on atypical life history could be broadly divided into three types: 1) gas vesicles and resting cells are not produced; 2) gas vesicles are formed but resting cells are not formed; 3) gas vesicles and akinetes are all formed (Stanier 1988; Karlsson Elfgren 2003; Hense 2006). Notably, the cyanobacteria species of the order Nostcoales are able to use gas vesicles to actively migrate between sediment and water layers (Abeynayaka et al. 2016). Numerous studies have been conducted to establish the optimal growth conditions of cyanobacteria (Robarts and Zohary 1987; Long et al. 2001; Nalewajko and Murphy 2001; Kwon 2014). Compared to the studies investigating the temperature and light conditions, only a few studies have investigated the nutrient salt condition for akinete germination. Thus, this study focused on the akinetes of the cyanobacteria *Dolichospermum circinale* isolated from Uiam Dam in the North Han River water system and the resting cells of the cyanobacteria *Microcystis aeruginosa* isolated near Ugok Bridge in the Nakdong River water system, and evaluated the morphology, germination process, germination rates, and growth in varying environmental conditions.

## Materials and methods

### Sample collection and isolation

**Fig. 1:**
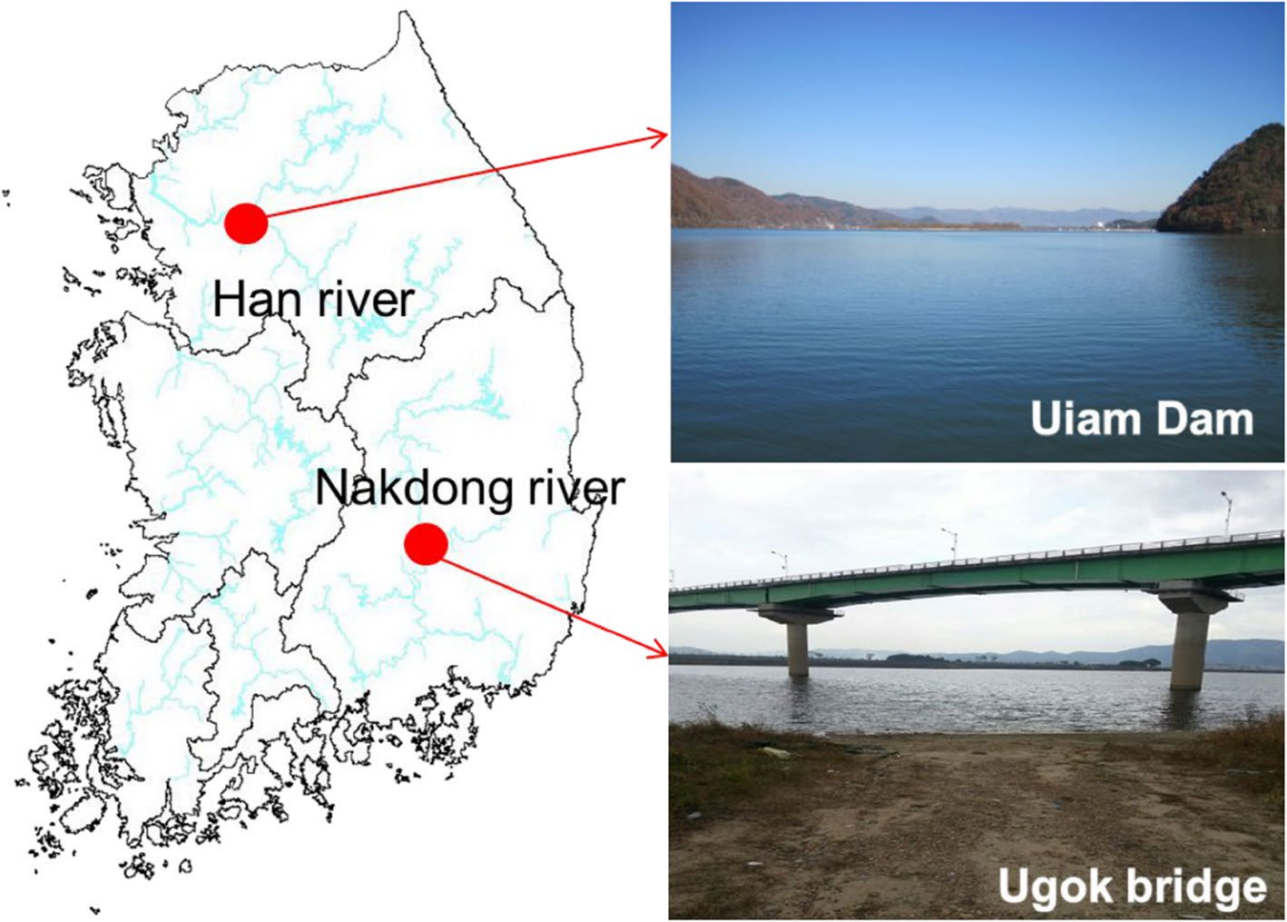
Study sites (akinetes were sampled at the Uiam Dam and resting cells were sampled at the Ugok bridge)

**Fig. 2:**
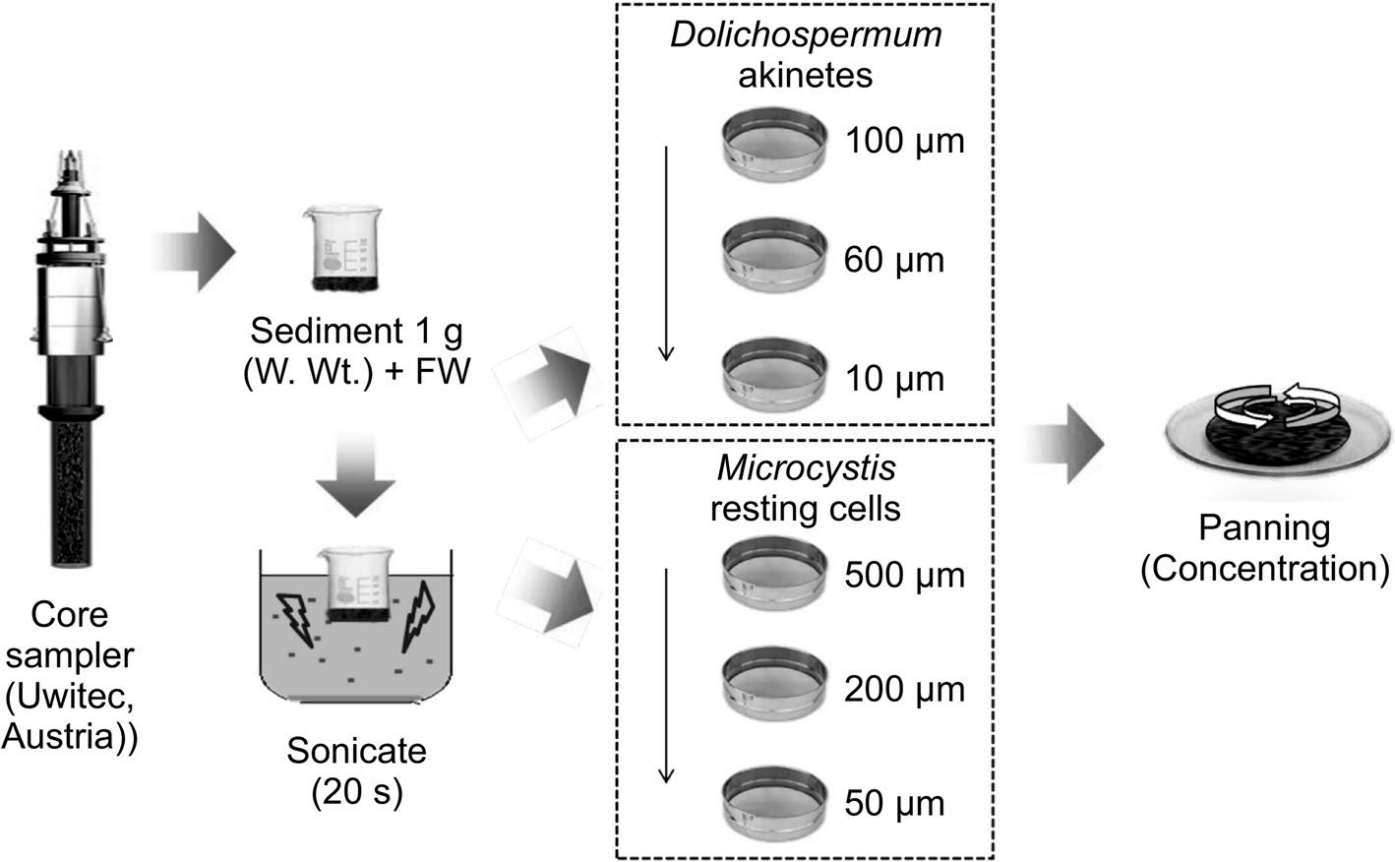
Separation of akinetes and resting cells using a core sampler. W. Wt. + FW

To isolate the akinetes of *Dolichospermum circinale* and resting cells of *Microcystis aeruginosa* from surface layer of the sediment, a sediment sample was collected using a core sampler (Uwitec, Austria) at Uiam dam (UD; N 37° 50′ 15.5″, E 127° 40′ 35.6″) in the North Han River water system and near Ugok bridge (UB; N 35° 37′ 20.4″, E 128° 23′ 30.6″) in the Nakdong River water system (Fig. 1, Table 1). The sample was placed in a 15 mL bottle and mixed thoroughly. The collected sample was then transferred to the laboratory, and 1 g (w/w) wet weight was stored in a refrigerator until further use. In the isolation pre-treatment of akinetes and resting cells, samples were filtered (0.2 μm) and suspended in sterilized water. The suspension was treated twice with ultrasound for 20 s using an ultrasonic device (JAC 4020 type, 60 Hz, 620 w, Ultrasonic, Korea). The pulverized suspension was sequentially filtered through 100, 60, and 10-μm nylon mesh to isolate akinetes, then through 500, 200, and 50-μm nylon mesh to isolate the resting cells. Following the panning method (Matsuoka and Fukuyo 2000), the filtrate was placed on a petri dish (12 cm in diameter), and from the top layer, the floating particles were isolated and mixed with filtered (0.2 μm) sterile water to a final volume of 10 mL. The final solution was stored in a dark brown glass bottle for storage at approximately 4℃ (Fig. 2).

**Table 1:** Sampling site information

Site	Location	Latitude (N)	Longitude (E)	Sample species
UD	Uiam-ri, Sindong-myeon, Chuncheon-si, Gangwon-do	37° 50′ 15.5″	127° 40′ 35.6″	Akinetes of *Dolichospermum circinale*
UB	Po-ri, Ugok-myeon, Goryeong-gun, Gyeongsangbuk-do	35° 37′ 20.4″	128° 23′ 30.6″	Resting cells of *Microcystis aeruginosa*

*UD* Uiam Dam, *UB* Ugok bridge

## Morphology and germination process

Stored samples (1 mL) were aliquoted to a Sedgwick–Rafter (SR) chamber, and following the microcapillary method and under an inverted microscope (Axiovert A1, ZEISS, Germany), the akinetes and resting cells were directly isolated individually and per colony, respectively. Filtered sterile water (0.2 mL) (originally from the site of sediment collection) was added to a 96-well plate, and the isolated akinetes (individual) and resting cells (colonies) were placed. The culture conditions were set at 25℃ and light intensity of 30–40 µmol m^−2^ s^−1^ (14 light: 10 dark) to maintain an optimal culturing level. The germination of akinetes and awakening of resting cells were monitored at a set time daily under 400 × and 1,000 × magnification using an inverted microscope, To elucidate the morphology of *D. circinale* akinetes and *M. aeruginosa* resting cells, an adhesive carbon tape was attached to the slide glass, to which the pre-treated cells were spread in wide dimensions, and cells were visualized using a field emission scanning electron microscope (FE-SEM) (MIRA 3, TESCAN, Czech Republic).

## Germination and growth rates in varying environmental conditions

To determine the germination and growth rates in each condition of temperature, light intensity, pH, and nutrient salt, each well of a 96-well plate was filled with 0.2 mL sterile Cyanobacteria (CB) medium, to which the *D. circinale* akinetes and the *M. aeruginosa* resting cells were placed individually and by colony, respectively. Cell cultures were performed in triplicate for each environmental condition (90 wells in total) for 14 days. The rate of *D. circinale* akinete germination was estimated based on the spore formation after the akinete germination in 24 h intervals and under an inverted microscope (Eclipse Ni, Nikon, Japan). The germination rate was calculated as the percentage of germinated akinetes against the total number of akinetes (%) applied in each experimental group, and the cumulative germination rate over 14 days was estimated. The rate of *M. aeruginosa* resting cell growth (μ) was calculated at each step of exponential increase of biomass in each environmental condition (Apha and WPCF 1995), and the maximal growth rate (μmax) was estimated using the Sigma plot (version 9.0, SPSS Inc.) using the following equation:

$$\mathrm\mu(\mathrm{day}^{-1})\:=\:\ln({\mathrm X}_2/{\mathrm X}_1)/({\mathrm T}_2\:-\:{\mathrm T}_1).$$

$${\mathrm X}_1:\;\mathrm{Chl}-\alpha\;\mathrm{concentration}\;\mathrm{at}\;\mathrm{the}\;\mathrm{onset}\;\mathrm{of}\;\mathrm{each}\;\mathrm{sampling}\;\mathrm{phase}.$$

$${\mathrm X}_2:\;\mathrm{Chl}-\alpha\;\mathrm{concentration}\;\mathrm{at}\;\mathrm{the}\;\mathrm{end}\;\mathrm{of}\;\mathrm{each}\;\mathrm{sampling}\;\mathrm{phase}.$$

$$({\mathrm T}_2-{\mathrm T}_1):\;\mathrm{Interval}\;\mathrm{of}\;\mathrm{each}\;\mathrm{sampling}\;(\mathrm{day}).$$

The standard environmental conditions were 25℃, light 30 µmol m^−2^ s^−1^, pH 7, and CB medium (N: 75 mg L^−1^, P: 7 mg L^−1^). To determine the rates of germination and growth for two isolated cyanobacteria species, the conditions were varied as follows: 5, 10, 15, 20, 25 and 30 °C for temperature; 5–12 for pH; 0, 5, 15, 30, 50 and 100 µmol m^−2^ s^−1^ for light. Lastly, regarding nutrient salts, four conditions were applied as the medium without nitrogen (N); the medium without phosphorous (P); the medium with both N and P; the medium with neither N or P (Table 2).

**Table 2:** Duplicated experiments at different temperature, light intensity, pH, and nutrient conditions

	**Temperature (℃)**	**Light intensity (μmol/m**^**2**^**s**^**1**^**)**	**pH**	**Nutrients (mg/L)**	**Analysis**
Exp. 1	5 10 15 20 25 30	30	9	CB medium (N: 75 mg/L, P: 7 mg/L)	Germination rate (%) Growth rate (%) Buoyancy
Exp. 2	25	30	5 6 7 8 9 10 11 12	CB medium	
Exp. 3	25	30	9	Distilled water − N, − P in CB + N, − P in CB − N, + P in CB + N, + P in CB	
Exp. 4	25	0 5 15 30 50 100	9	CB medium	

*Exp* experiment, *CB* Cyanobacteria medium

## Floating characteristics according to vertical migration speed

To examine the floating characteristics of akinetes and resting cells, 5 mL samples were extracted using a pipette from the upper and lower layers on the upper part of the cylinder at set intervals (1, 3, 5, 10, 15, 20, 30, 40, and 60 min) on the 14th day of culture, i.e., the last day of the experiment. The difference in the Chl-*a* concentration ratio across the samples was analyzed, through which the vertical migration time (T50) was estimated for akinetes and resting cells to measure the precipitating and floating characteristics, using the following equation (Titman 1975; Wu et al. 2020).

$$\mathrm{Floating}=\frac{\mathrm d}{{\mathrm T}_{50}}$$

$$\mathrm d:\;\mathrm{distance}\;\mathrm{of}\;\mathrm{migration}\;(\mathrm{cm}).$$

$${\mathrm T}_{50}:\;\mathrm{time}\;\mathrm{to}\;\mathrm{difference}\;\mathrm{in}\;\mathrm{Chl}-\alpha\;\mathrm{concentration}\;\mathrm{by}\:\geq\:50\%\;\mathrm{between}\;\mathrm{upper}\;\mathrm{and}\;\mathrm{lower}\;\mathrm{layers}\;(\min).$$

## Results

### Akinete morphology and germination process of Dolichospermum circinale

**Fig. 3:**
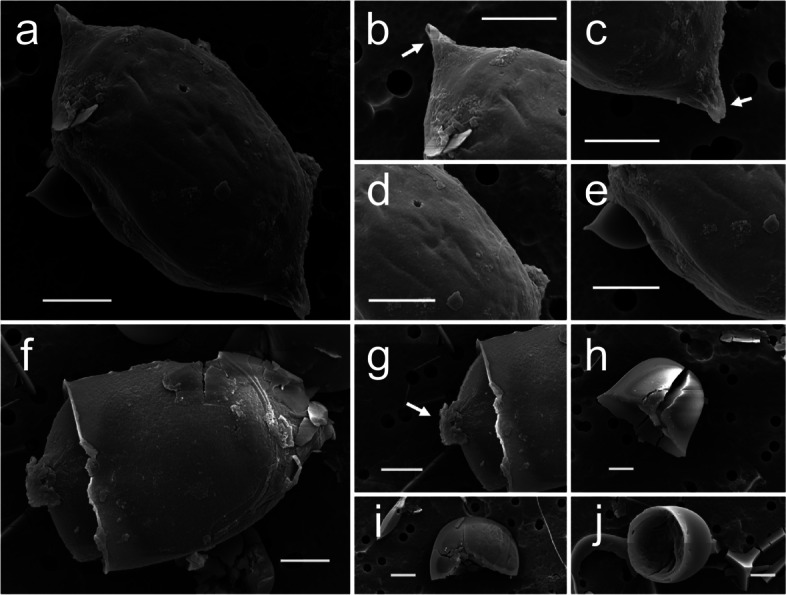
Akinete scanning electron microscopy (SEM) images of *Dolichospermum circinale*. **a** Akinene’s typical morphology **b**, **c** akinete’s left and right warts, **d**, **e** akinete’s upper part and side images, **f** akinete’s germination and exuviation, **g** a connecting link with a trophocyte following exuviation, **h**, **i**, **j** various types of akinete shells. Scale bar = 5 µm

The akinete morphology varied from a long-ellipsoidal type to a short-ellipsoidal (Fig. 3; a, d, e, h, i, j), and the width and length of akinetes generally ranged from 15—22 μm and 12—16 μm, respectively (Fig. 3a). In general, warts were found on the surface of akinetes (Fig. 3; b and c) although not all akinetes displayed warts (Fig. 3; h and i). The protruding warts on either ends of the cells that could be observed even under a light microscope are presumed to be the trace of the connection across the single cells of the order Nostocales (Fig. 3; a, b, and c). In addition, as the akinete morphology was being examined, a form of spore germination was found, and warts could be simultaneously observed on vegetative cells with exuviation (Fig. 3; f and g).

**Fig. 4:**
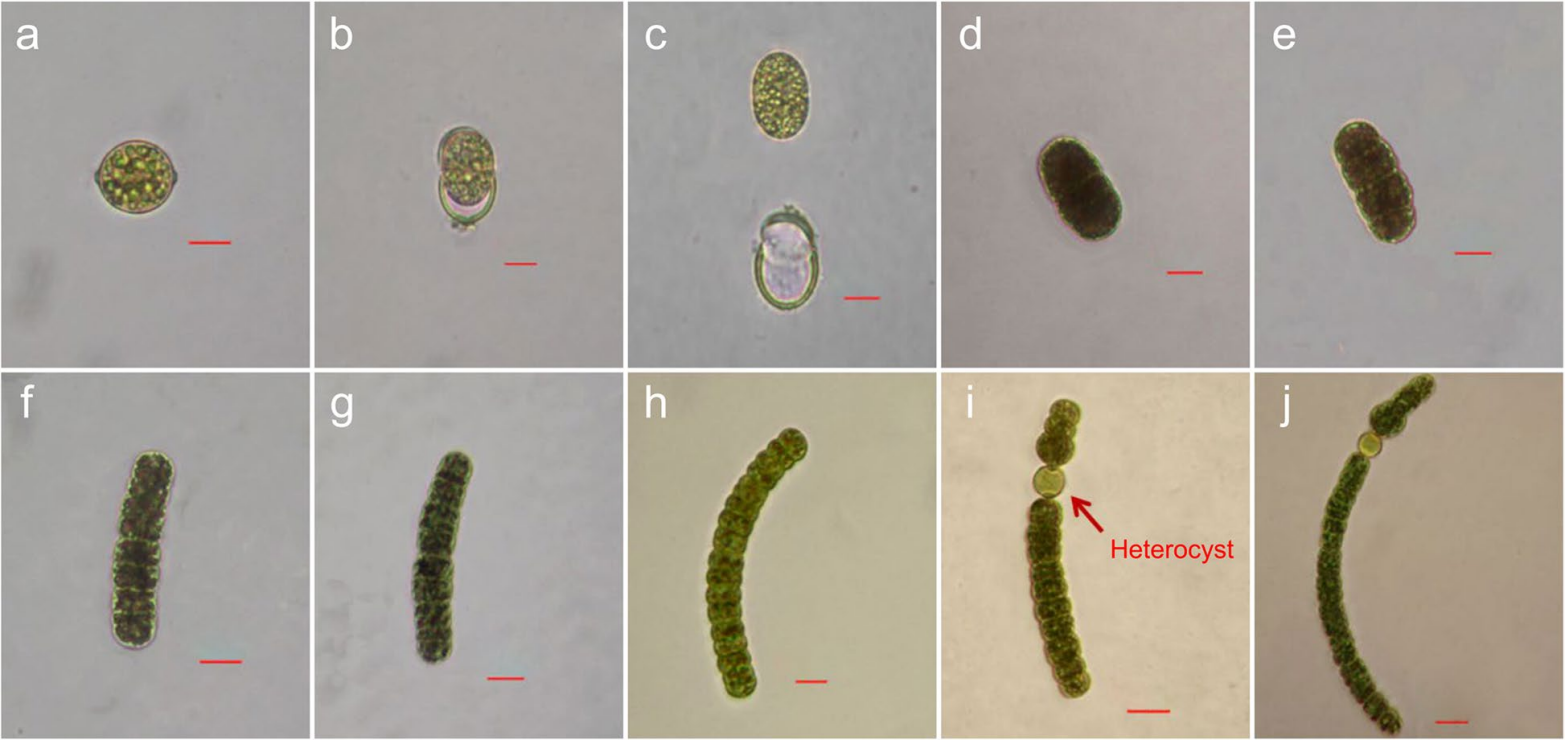
*Dolichospermum circinale* akinete germination. Scale bar = 10 µm

For more accurate analyses of the morphology and germination process, the microscopic observation was performed in one day intervals for a period of 10 days. The *D. circinale* akinetes isolated from the sediment layer exhibited an ellipsoidal shape, light green in color with 15–18 μm width and 13–16 μm length, and short warts of 0.1–0.2 μm at each end of akinete (Fig. 4a). On day two of culture, a phenomenon of exuviation by which the germinating akinete penetrated out of the cell wall was observed, while the cell walls surrounding the spore could be examined in detail (Fig. 4b). On day three of culture, akinete germination was observed with the completion of exuviation at cell walls, and after the germination, empty envelopes were observed. The germinated cells exhibited an ellipsoidal shape with 21–23 μm width and 15–16 μm length (Fig. 4c). Compared to the size of akinetes before germination, the size of those escaping the spore was larger by length, which could be because cell growth had already begun in the germination process. On the day four of culture, the spore turned dark in color and approached a greenish black with 29–30 μm width and 15–16 μm length (Fig. 4d). On day five of culture, the color was lighter than that on the 4th day with the cells exhibiting clearly visible nodes (Fig. 4e). On day six of culture, the width and length of the cells were 42–44 μm and 10–11 μm, respectively, indicating an increase in width but a decrease in length (Fig. 4f). After the 6th day of culture, the proliferating cells formed a trichome, whose length continued to increase but whose width was maintained within 10–12 μm (Figs. 4g–h). The size of each single cell continuously increased, and on the 8th day of culture, the cells approached a lengthy connected trichome at 65–67 μm width and 10 μm length (Fig. 4h). On the 9th day of culture, the length of each single cell further decreased (from 10 to 7–8 μm) as the length of the trichome increased, and heterocyte, a critical cellular morphology, was observed at 6–8 μm diameter, which was smaller than the size of the surrounding vegetative cells (Fig. 4i). On the 10th day of culture, proliferation of vegetative cells on the upper and lower parts of heterocyte was observed, and the trichome size further increased to 120 μm width and 7 μm length (Fig. 4j).

### Microcystis aeruginosa awakening

**Fig. 5:**
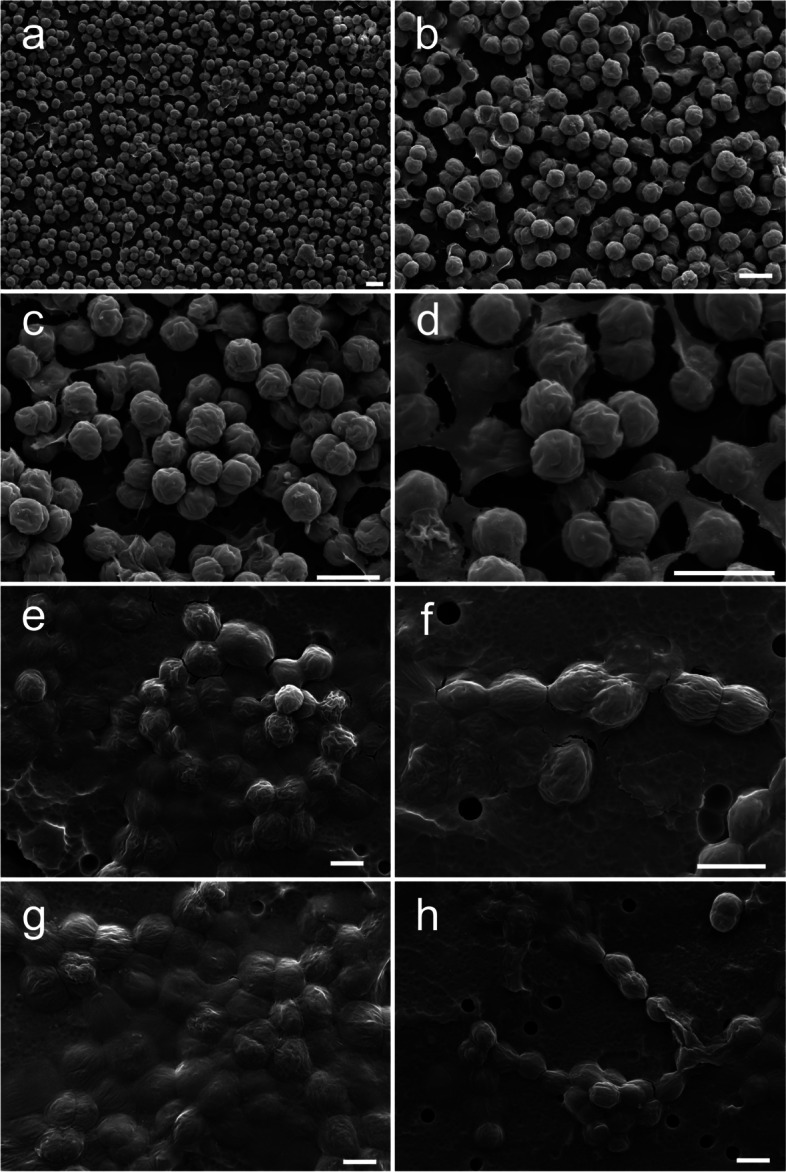
Scanning electron microscopy (SEM) images of *Microcystis aeruginosa*. **a**–**d** colony of *M. aeruginosa*, **e**–**h** resting cells *of M. aerginosa.* Scale bar = 5 µm

Analyzing the microstructure of *M. aeruginosa* with colony formation indicated that the mean size of single cells was approximately 2 μm, and one to two cells constituted a bundle toward colony formation (Fig. 5; a-d). The resting cells isolated from the sediment layer had a microstructure with cells surrounded by mucus that connected the cells rather than the colony formation (Fig. 5; e–h).

**Fig. 6:**
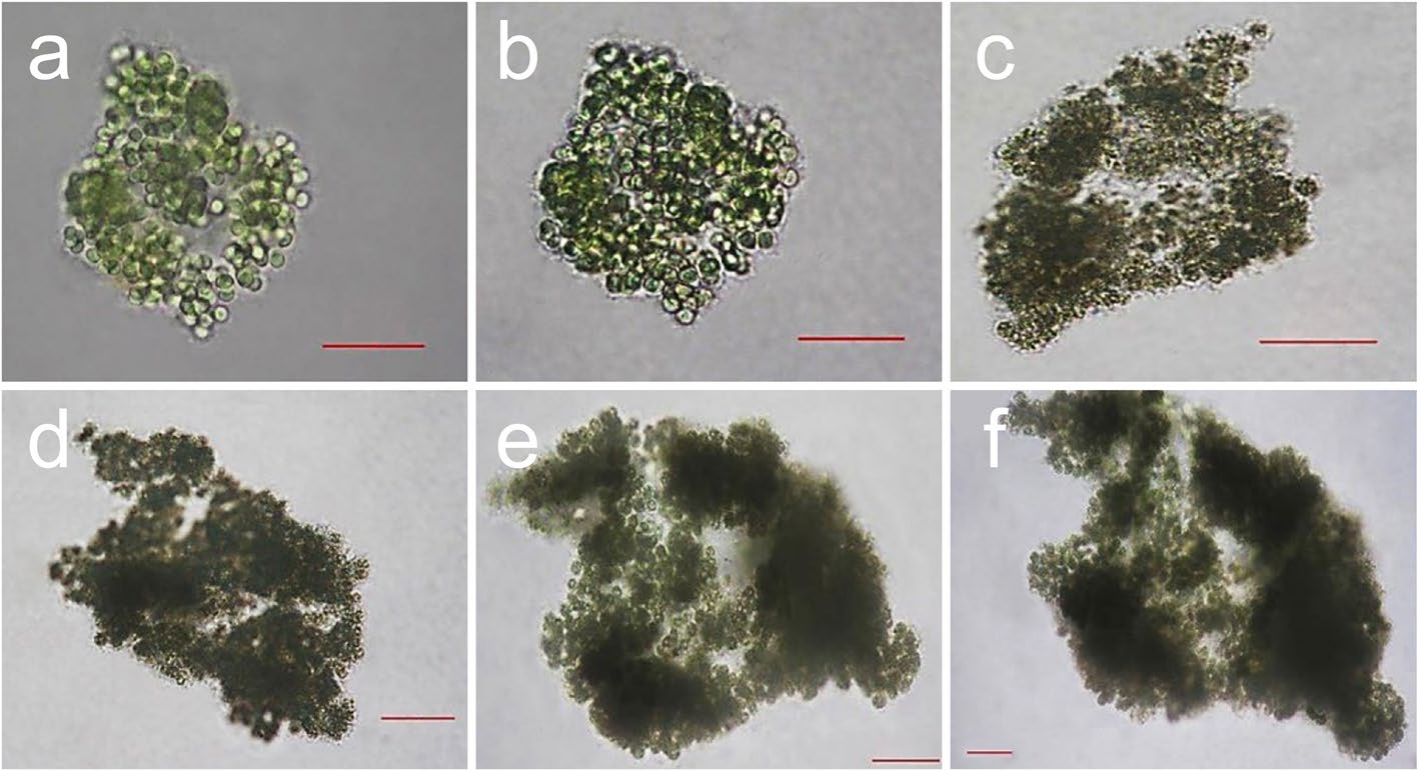
*Microcystis aeruginosa* resting-cell awakening. Scale bar = 30 µm

For accurate analyses of the morphology and germination process, microscopic observation was performed in one day intervals for a period of six days (Fig. 6). The *M. aeruginosa* colonies isolated from the sediment layer were light green in color, with areas of 50–100 μm (Fig. 6a). On the 2nd day of culture, the colonies were dark green in color without a significant change in size (Fig. 6b). On the 3rd day of culture, the cell density substantially increased, with an area of 85–130 μm, showing an increase in size. The color on the exterior had also begun to turn greenish black (Fig. 6c). On the 4th day of culture, the external color approached black, and the area was between 115–165 μm. On the 5th and 6th days of culture, the colonies showed an increase in area and color intensity so that the levels were over twofold higher on the 6th day compared to the initial states; the area ranged between 190–330 μm with substantially higher cell density (Figs. 6d-e).

### Growth characteristics in varying environmental conditions

**Fig. 7:**
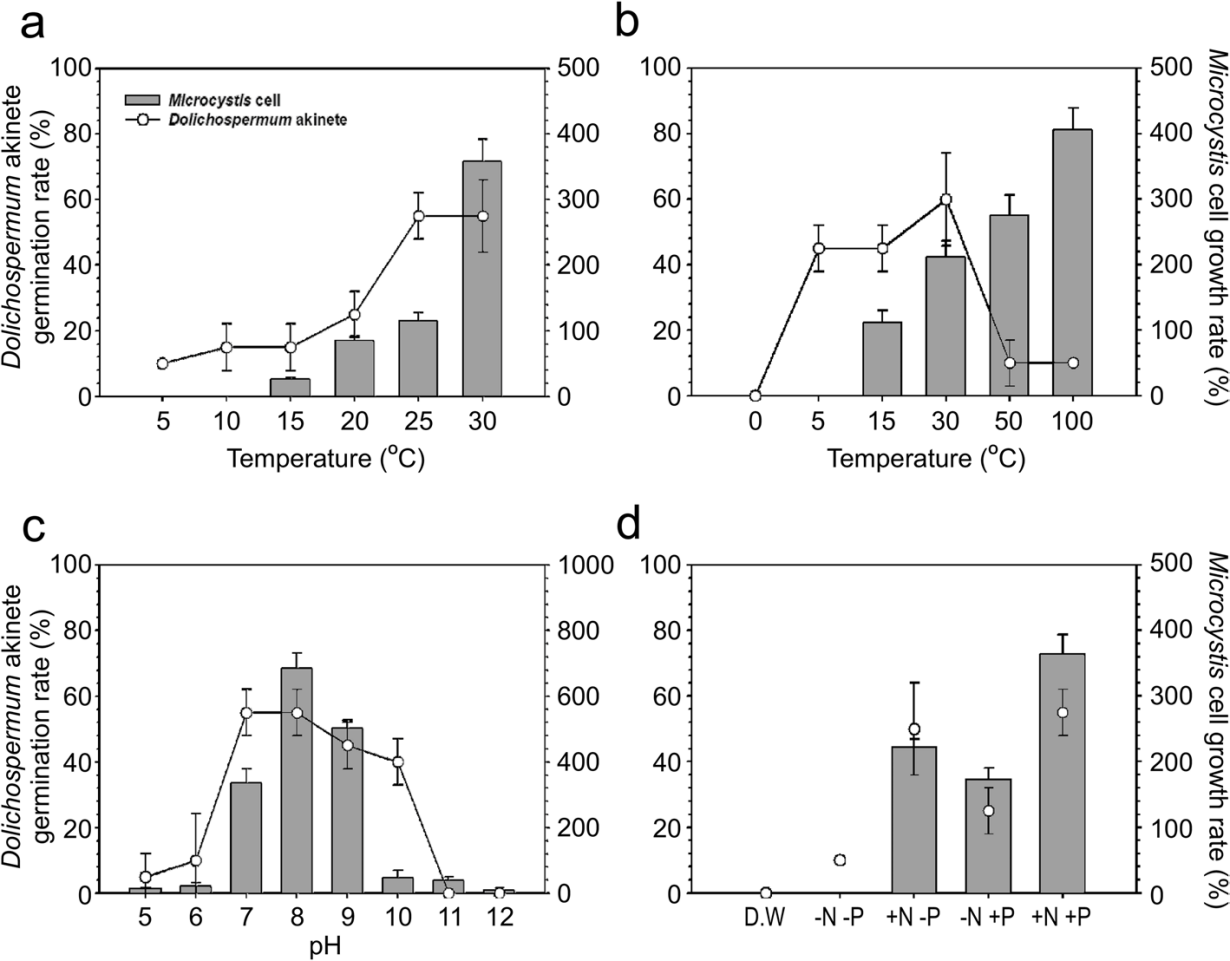
Germination and growth rates of akinetes and resting cells under various conditions

The *D. circinale* akinete germination and *M. aeruginosa* awakening rates were determined in varying environmental conditions. The result indicated that the rate of akinete germination was the lowest (10%) at 5℃, and the rate increased as the water temperature increased. The germination rate was ≥ 20% at 20℃ and ≥ 60% at 25℃ and 30℃, revealing a > sixfold increase compared to the rate at 5℃ (Fig. 7a). The resting cells did not exhibit growth in low temperature conditions at 5℃ and 10℃, as the cells died. The rate of cell growth was 30% from 15℃ and reached ≥ 100% at 20℃ and 25℃. The highest rate of *M. aeruginosa* growth was observed at 30℃, which was ≥ 350% (Fig. 7a). The floating characteristics according to temperature demonstrated that the two species of cyanobacteria both showed floating at temperatures ≥ 20℃, at which the rates of germination and growth increased, whereas precipitation was observed at temperatures ≤ 15℃ (Table 3). Analyzing the germination rate of akinetes and growth rate of resting cells according to light conditions indicated that the resting state of the cells continued in the condition without light, then in the light condition of 5 μmol m^−2^ s^−1^, the rate of akinete germination was considerably high (approximately 50%) and in the light condition of 30 μmol m^−2^ s^−1^, the rate of akinete germination was the highest (60%). From 50 μmol m^−2^ s^−1^ and onwards, the rate rapidly decreased to 10% (Fig. 7b). The resting cells showed the first signs of growth in the light condition of 15 μmol m^−2^ s^−1^. The growth rate increased as the light intensity increased to reach the highest rate of ≥ 400% in 100 μmol m^−2^ s^−1^ (Fig. 7b). The akinetes showed floating in the conditions of 5–30 μmol m^−2^ s^−1^ with high germination rates and the resting cells exhibited floating in the 15–100 μmol m^−2^ s^−1^ conditions (Table 3). The germination rate of akinetes and growth rate of resting cells according to pH indicated optimal levels of germination and growth at pH 7–9. The germination rate was approximately 60% at pH 7 and 8, and the growth rate was high, ≥ 700%, at pH 8. In contrast, as the pH approached strong acidity or basicity, the germination and growth rates both decreased (Fig. 7c). A general trend of floating was observed at pH 7–9 with relatively high rates of germination and growth, but precipitation was observed in all other pH conditions (Table 3). The akinete germination and resting cell awakening in varying nutrient salt conditions indicated that neither was observed in distilled water. In the medium without N and P, the rate of akinete germination was approximately 10% although no awakening of resting cells was detected, and in the medium with both N and P, the growth rate of resting cells was ≥ 350% and the germination rate of akinetes was high, ≥ 50%. In contrast, the rates of germination and growth both decreased by more than half in the medium without N, but in the medium without P, the germination rate of akinetes was high, ≥ 50%, while the growth rate of resting cells was ≥ 200% (Fig. 7d). This suggested that N had a stronger impact on akinete germination than P. Regarding floating characteristics, the cells in the medium with N showed floating but those in the medium without N or distilled water showed precipitation (Table 3).

**Table 3:** Duplicated experiments at different temperature, light intensity, pH, and nutrient conditions on the buoyancy of *Dolichospermum circinalils* and *Microcystis aeruginosa*

	**Temperature (℃)**	***Dolichospermum circinalils***	***Microcystis aeruginosa***
Exp. 1	5 10 15 20 25 30	□ □ □ ■ ■ ■	□ □ □ ■ ■ ■
**Light intensity (μmol/m2·s)**			
Exp. 4	0 5 15 30 50 100	□ ■ ■ ■ □ □	□ □ ■ ■ ■ ■
**pH**			
Exp. 2	5 6 7 8 9 10 11 12	□ □ ■ ■ ■ ■ □ □	□ □ ■ ■ ■ □ □ □
**Nutrients (mg/L)**			
Exp. 3	Distilled water − N, − P in CB + N, − P in CB − N, + P in CB + N, + P in CB	□ □ ■ □ ■	□ □ ■ □ ■

□: Negative buoyancy; ■: Positive buoyancy; *CB* Cyanobacteria medium

## Discussion

Environmental factors such as light, temperature, nutrient salts, pH and oxygen are associated with akinete germination and resting cell awakening (Dok and Hart 1997; Ståhl-Delbanco et al. 2003). Each species of cyanobacteria has unique optimal environmental conditions for germination and growth (Huber 1985; Mischke 2003). Water temperature is a key environmental factor that promotes cyanobacteria blooms, and many species of cyanobacteria show a preference for high water temperatures (Robarts and Zohary 1987). For instance, *Microcystis aeruginosa*, *M. wesenbergii*, *Dolichospermum spiroides*, *Raphidiopsis raciborskii*, *Nodularia spumigena* and *Aphanizomenon flos-aquae* show high emergence mainly in summer with high water temperature (Kanoshina et al. 2003; Hong et al. 2006; Imai et al. 2009). The increased temperature of the waterbody and sediment layer in spring and early summer induces cyanobacteria akinete germination and resting cell awakening, while facilitating the metabolic activities of germinating cells toward exponential growth (Paerl 1988). Thus, water temperature is an important factor that facilitates the metabolic activities of cyanobacteria (Robarts and Zohary 1987). This study demonstrated that the germination of *D. circinale* akinetes was induced at the water temperature of 25 °C–30 °C and that the growth of *M. aeruginosa* resting cells rapidly increased at 30℃, suggesting that the rates of germination and growth were high as the high water temperature was maintained. However, at ≤ 15℃, the two cyanobacteria species showed low rates of germination and growth, and in the conditions of 5℃ and 10℃, no resting cell growth was detected. Certain species of cyanobacteria such as *Anabaena cylindrica* and *Aphanizomenon flos-aquae* grow at low water temperatures (Li et al. 1997; Tsujimura et al. 2001); and *D. mendotae* akinetes showed a markedly high rate of germination at low temperatures ≤ 10℃ (Kim et al. 2005). This suggested that, despite a comparatively low rate of germination at low water temperatures, the level of germination could still contribute to the proliferation of vegetative cells, which coincided with the results in other previous studies. *Dolichospermum mendotae*, *Nodularia spumigena* and *Raphidiopsis raciborskii* showed the highest akinete germination rates in the range of 20 °C–25℃ (> 40%), while the rate decreased at low temperatures ≤ 10℃ (< 15%) (Huber 1985; Kravchuk et al. 2002). *M.* aeruginosa and M*. wesenbergii* showed a rapid population increase with a sudden rise in temperature to ≥ 25℃, which was attributed to the growth within the sediment layer (Yamamoto and Nakahara 2009). *D. mucosum* akinetes at low water temperatures (5 °C–8℃) showed a limited rate of germination ≤ 10%, whereas the rate increased by ≥ 60% in an environment of relatively high water temperatures (14 °C–23℃) (Tsujimura 2004). Hence, *D. circinale* germination and *M. aeruginosa* awakening could be induced at water temperatures of ≥ 25℃, which would have a direct influence on cell proliferation. Consequently, in the stepwise process from akinete germination to resting cell awakening, the temperature acts as a trigger, and the water temperature of a specific range (25 °C–30℃) can increase the physiological activities of akinetes and resting cells. This is presumed to influence the response time from triggering that causes re-entry to germination and optimal growth so as to determine the range of temperature for germination and growth (Roelofs and Oglesby 1970; Kuoyu et al. 2004). Light is another crucial environmental factor that influences cyanobacterial germination and growth (Matthijs et al. 1998; Khatoon et al. 2018). For instance, the germination rate of *Nodularia spumigena* akinetes was the highest at the light intensity of 15–50 μmol m^2^ s^1^ but no germination occurred in the dark condition without light (Huber 1985). For many biological species, light induces the circadian or seasonal lifecycle, and induces the germination and growth of cyanobacteria, acting as a trigger (Huber 1985; Dok and Hart 1997; Barbiero and Kann 1994; Baker and Bellifemine 2000). The akinetes of certain cyanobacteria species have been reported to germinate in conditions without light (Neely-Fisher et al. 1989); however, light is an essential condition for the germination and growth of most cyanobacteria akinetes and resting cells (Agrawal 2009). In general, high light intensity is required for cyanobacteria germination and growth, which may still present adequate levels, even at low intensity (Huber 1985; Dok and Hart 1997). The two isolated cyanobacteria species in this study demonstrated no germination or growth in the condition without light, but the germination of *D. circinale* could be induced in the condition of lowest light intensity of 5 μmol m^2^ s^1^, and the onset of growth of *M. aeruginosa* was observed from 15 μmol m^2^ s^1^, despite the absence of growth in the condition of minimal light intensity of 5 μmol m^2^ s^1^. The growth of *M. aeruginosa* was shown to increase as the light intensity increased, whereas the germination of *D. circinale* rapidly decreased from the light intensity ≥ 50 μmol m^2^ s^1^, which indicated a variation in the growth pattern between the two species. As can be seen, an increase in light intensity or a condition of minimal light intensity could promote the growth of akinetes and resting cells, and the rate of growth could increase in a specific range of light intensity (Yamamoto 1976; Agrawal 1984, 1986). Another known environmental factor that is critical for cyanobacteria growth and akinete germination is the pH (Agrawal and Misra 2002; Lopez-Archilla et al. 2004). In this study, pH ≤ 6 and pH ≥ 11 led to low rates of *D. circinale* germination ≤ 10%, and pH 7–10 led to high germination rates (≥ 40%) with optimal growth. For *M. aeruginosa*, pH ≤ 6 and pH ≥ 10 led to growth rates < 50%, and the conditions of pH 7–10 led to high growth rates ≥ 300%. At pH 8, in particular, the rate of cell growth approached 800%. These results coincided with those of previous studies. Other species such as *Microcystis aeruginosa*, *Anabaena vaginicola*, *Anabaena cylindrica*, *Westiellopsis prolifica*, *Anabaenopsis arnoldii* and *Nostochopsis lobatus* also showed the highest rates of germination and growth at pH 7–8 (Agrawal and Misra 2002; Reddy 1983; Huang et al. 2019). In an acidic condition, phaeophytin is degraded (Brock 1973) with a potential reduction of cell viability or damage on the akinete cell wall (Agrawal 2009). Meanwhile, high pH could alter the permeability of cell membrane (Holm-Hansen 1963), and the reduced absorption of anions at pH ≥ 11 could inhibit the cellular physiological activities and cause insolubility of carbon dioxide to prevent photosynthesis (Rai and Pandey 1981). Consequently, we suggest that each cyanobacteria species has an optimal pH condition for adequate growth and that the rates of germination and growth could be optimized by maintaining such optimal conditions (Agrawal 2009; Agrawal and Misra 2002). For the germination and growth of cyanobacteria, different nutrient salts may play different roles (Nalewajko and Murphy 2001), and notably, N and P are known to have an effect on the germination and growth of cyanobacteria (Huber 1985; Dok and Hart 1997; Agrawal and Misra 2002; Rai and Pandey 1981). As an example, the akinetes of different cyanobacteria species; *Anabaena fertilissima*, *Anabaena variabilis,* and *Nostoc linckia*, showed different rates of germination and growth in previous studies testing the conditions of P and N depletion or addition (Yamamoto and Nakahara 2005; Reddy 1983). The population increase of *Microcystis* in the water layer could increase the concentration of P, and the relatively high concentration of N in the sediment layer could contribute to the formation of colonies of *Microcystis* resting cells in the sediment (Ståhl-Delbanco et al. 2003; Xie et al. 2003). In this study, the rate of *D. circinale* akinete germination was 10% in the condition without N and P but the rate was high, ≥ 50%, in the condition with both N and P. For *M. aeruginosa*, no growth was observed in the condition without N and P, and the rate of growth was the highest in the condition with both N and P. Notably, the germination and growth rates were higher in the condition without P than in the condition without N for both species, which suggested a slightly higher influence of N than P on the growth of the two cyanobacteria species. For the two isolated species in this study, the N from NH_4_N and P from phosphate were shown to be the critical factors that contribute to the germination and growth. As a limiting element of algal biosynthesis, N allows algal growth via its metabolism. The N from NH_4_N is one of the most essential elements primarily required for algae; the N dissolved in the waterbody is reduced to ammonia and used in the synthesis of amino acids (Graham et al. 2010). The germination rate of *Westiellopsis prolifica* akinetes showed an over fivefold increase in the condition with N or P compared to the condition without N or P (Agrawal and Sharma 1994). The germination rate of *Nodularia spumigena* akinetes also increased by ≥ 68% as the concentration of P increased (Huber 1985). In contrast, the addition or depletion of N did not have a significant effect on the germination of *Anabaena cylindrica* and *Nostoc* sp. akinetes (Yamamoto 1976; Sutherland et al. 1985), whereas Huber (Huber 1985) reported an increase in germination rate by 70%–80% for *Nodularia spumigena* akinetes in the condition of N addition. In this study, *D. circinale* akinetes showed a high rate of germination even in the condition of low phosphate salt concentration, based on which phosphate salt was presumed not to be an essential factor for germination. The results implied that, to control the eutrophication of freshwater ecosystems, the management of N in addition to P was required. The results in this study regarding the rates of cyanobacteria germination and growth in varying environmental conditions collectively suggested that the optimal conditions to allow *D. circinale* germination were water temperature 25 °C–30 °C, light intensity 5–30 μmol m^2^ s^1^, pH 7–10, and adequate levels of N and P, while the optimal growth conditions for *M. aeruginosa* were water temperature 30℃, light intensity 50–100 μmol m^2^ s^1^, pH 8–9, and adequate levels of N and P. The investigation of the germination and growth of two cyanobacteria species isolated from the sediment layers of the Han River and Nakdong River water systems revealed that it is necessary to conduct in-depth analyses of the life history of cyanobacteria regarding germination and growth in sediment layers and furthermore to predict algal blooms in future and develop appropriate response measures. In addition, studies to deepen the understanding of the distribution and development of cyanobacteria akinetes and resting cells should be conducted with a focus on specific water bodies, and respective management policies should be developed.

## Conclusions

The results of this study are expected to be important for the management of harmful algae in the Han River and Nakdonggang River water systems. In particular, *D. circinale* and *M. aeruginosa* which have been dominant as drinking water sources in the North Korean river and Nakdonggang River water systems for the past few years, have received social attention. Akinete and resting cells in sediments affect germination and growth due to a combination of physical and chemical factors such as water temperature and nutrient salt concentration in the light water body, i.e. the most essential requirement for understanding the outbreak of cyanobacteria is how species-specific life is working in the ecosystem. Therefore, it is necessary to improve the study of life history mechanisms including the occurrence and extinction of cyanobacteria through the accumulation of basic data through this study and various aspects of research.

## Data Availability

The datasets used and/or analyzed during the current study are available from the corresponding author on reasonable request.

## Abbreviations

CB: Cyanobacteria
FE-SEM: Field emission scanning electron microscope
SR: Sedgwick–Rafter
UB: Ugok bridge
UD: Uiam dam
